# Odors in space

**DOI:** 10.3389/fncir.2024.1414452

**Published:** 2024-06-24

**Authors:** Olivia McKissick, Nell Klimpert, Jason T. Ritt, Alexander Fleischmann

**Affiliations:** ^1^Department of Neuroscience and Carney Institute for Brain Science, Brown University, Providence, RI, United States; ^2^Carney Institute for Brain Science, Brown University, Providence, RI, United States

**Keywords:** hippocampus, piriform cortex (PC), virtual reality, cognitive map, olfaction, entorhinal cortex, learning and memory

## Abstract

As an evolutionarily ancient sense, olfaction is key to learning where to find food, shelter, mates, and important landmarks in an animal’s environment. Brain circuitry linking odor and navigation appears to be a well conserved multi-region system among mammals; the anterior olfactory nucleus, piriform cortex, entorhinal cortex, and hippocampus each represent different aspects of olfactory and spatial information. We review recent advances in our understanding of the neural circuits underlying odor-place associations, highlighting key choices of behavioral task design and neural circuit manipulations for investigating learning and memory.

## An ancient and important cognitive ability: odor-space integration

Across evolutionary distant species, animals have harnessed their sense of smell to navigate and survive in diverse habitats. This enduring significance of olfaction is reflected in the remarkable structural similarity of olfactory neural circuits. For example, the three-layered cytoarchitecture of the mammalian olfactory cortex resembles the pallial structures of amphibians and reptiles and distinguishes the mammalian olfactory cortex from the 6-layered neocortical areas for vision, hearing, or touch (1–6). This review will focus on rodents, though there are parallels to research in other organisms, notably insects (7–10).

Foraging for food is one of many odor-driven tasks that a rodent must perform to survive. Consider a mouse searching for something to eat: while vision and other senses may contribute to the search, olfaction is key to, say, finding seeds buried in a forest (11), for example by following odor plumes to their source (12, 13). It may further benefit the mouse in the long term to remember the location of this seed stash. By piecing together a map of scents at the stash and along the way home, the mouse can plan for more efficient future foraging. This ability to integrate spatial and olfactory information may in fact be more central to olfactory system evolution than other tasks such as odorant discrimination (9). Also critical is an ability to update this information as the environment changes; if the stash is eaten, the mouse will have to expand its map with new food sources.

In this review, we illustrate neural mechanisms underlying the integration of odor and space. We first discuss candidate multi-area circuit structures. We then review recent findings that support the functional role of these circuits revealed through cleverly designed behavioral tasks combined with neural recording and manipulation. We discuss research into changes in representations during learning and summarize ongoing technological advances that will help address key open questions.

## Neural circuits for odor-space coding

Several regions of the olfactory system, including the olfactory bulb (OB), anterior olfactory nucleus (AON), and piriform cortex (PCx), connect directly to higher-level spatial processing and memory areas such as the hippocampus (HPC) and lateral entorhinal cortex (LEC). With both bottom-up and top-down inputs, each area is proposed to play an important role in the transfer of odor information from the periphery and its integration with contextual and spatial information. We will describe each area’s key cell types, and how inter-areal connectivity is proposed to underlie function.

### Olfactory input: from periphery to cortex

**Figure 1:**
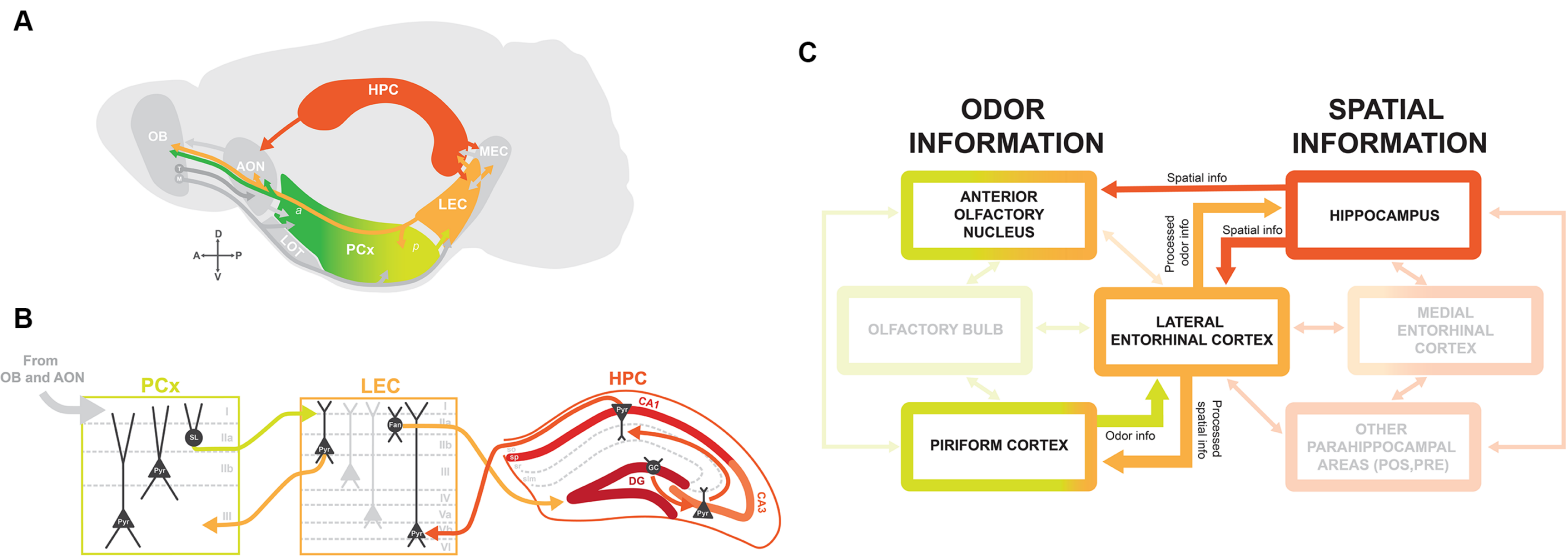
Brain circuitry for integrating olfactory-spatial information. **(A)** Schematic of brain regions involved in odor-place coding, with arrows indicating connections between brain regions. The lateral olfactory tract (LOT) traditionally defines the boundary between anterior (*a*) and posterior (*p*) PCx. OB, olfactory bulb; M, mitral cells; T, tufted cells; AON, anterior olfactory nucleus; PCx, piriform cortex; LEC, lateral entorhinal cortex; MEC, medial entorhinal cortex; HPC, hippocampus. **(B)** Circuitry between PCx, LEC, and HPC with main excitatory projection cell types. Information from olfactory areas OB and AON is relayed to PCx via layer I. PCx layer IIa semilunar (SL) cells project to LEC layer I, and LEC projects back to PCx via layer IIb CB+ pyramidal cells (Pyr). LEC projects to dentate gyrus granule cells (GC) from layer IIa RLN+ fan cells (Fan) via the perforant pathway. Information received from LEC is routed through HPC via the trisynaptic circuit, from granule cells to pyramidal cells in CA3 and CA1, and back to LEC to deep layer Vb pyramidal cells. **(C)** Flow chart indicating the flow of odor and spatial information from the main brain regions implicated in odor-place coding: AON, PCx, LEC, and HPC. Odor information from PCx and spatial information from HPC are directed to LEC. The LEC then relays processed odor information to HPC and processed spatial information back to PCx. The AON receives direct input from HPC and has been suggested as an alternate integrator of odor and spatial information.

Odor information detected by olfactory sensory neurons (OSNs) in the olfactory epithelium is relayed to the olfactory cortex via mitral and tufted cells in the OB. Areas of the olfactory cortex, which include the AON, PCx, and LEC, receive olfactory input via molecularly distinct subtypes of mitral and tufted cells that preferentially project to AON, PCx, or LEC (Figure 1A) (14–18).

PCx is divided into molecularly and functionally segregated anterior piriform (aPCx) and posterior piriform (pPCx), with their boundary traditionally defined by the termination of the lateral olfactory tract (LOT) (Figure 1A) (19). There are major differences in circuitry between the aPCx and pPCx: aPCx receives more input from mitral and tufted cells in OB and has more bidirectional connections with AON, while pPCx is more connected to higher order areas like LEC and the cortical amygdala (COA) (16, 18, 20–22). The circuitry is in line with functional segregations between aPCx and pPCx, as aPCx is thought to primarily encode odor identity, and pPCx plays a more important role in the association and encoding of context and spatial position (23–29).

The PCx is organized in a trilaminar structure: an axonal layer I where afferent inputs from OB and AON are received, a dense layer II containing semilunar and pyramidal cells, and a deep layer III containing mostly pyramidal cells (Figure 1B). Output projections from PCx are largely segregated by layers, as molecularly distinct layer IIb cells and layer III pyramidal cells preferentially project back to OB and to frontal cortical regions, while layer IIa semilunar cells preferentially project to LEC and COA (19–22). Furthermore, projections of deep layer cells to frontal regions are segregated along the anterior/posterior axis, with projections from aPCx to orbitofrontal cortex (OFC) and from pPCx to medial prefrontal cortex (mPFC), and these frontal areas play unique roles in olfactory learning and representations of odor value (21, 30–32). The neural circuitry and cytoarchitecture of the olfactory cortex therefore correspond to the differential routing of olfactory information to regions throughout the brain.

### Space and context: hippocampal formation

The hippocampal formation plays a key role in spatial learning and memory, as place cells, grid cells, and head direction cells selectively encode an animal’s position in their environment (33). HPC has strong bidirectional connections with the entorhinal cortex and receives other parahippocampal inputs and projections to higher-order areas like the amygdala and striatum (34). For the scope of this review, we focus primarily on its connection with the olfactory cortex via bidirectional connections with LEC. Although there is a large degree of intrinsic connectivity within the hippocampus, the hippocampal circuit is classically defined by the ‘trisynaptic circuit’: HPC receives input from the entorhinal cortex via dentate gyrus (DG) granule cells, transfers this information through the HPC via synaptic connections between CA3 and CA1 pyramidal cells, and then routes information back to the entorhinal cortex both directly and indirectly via the subiculum (Figure 1B) (35).

The connectivity patterns of HPC vary along the dorsal/ventral axis. Dorsal HPC receives most of its input from the entorhinal cortex. By contrast, ventral HPC is more connected to amygdalar, limbic, and olfactory regions. There is some evidence that these differences in circuitry relate to differences in the functional role of dorsal vs. ventral HPC, with more spatial coding in dorsal HPC and a larger role of ventral HPC in learned emotional behaviors (36–39). This functional segregation, however, remains an open question. It is important to note that recording studies in the HPC tend to focus on the dorsal sections, including the ones to be discussed later in this review.

### Odor and spatial integration: lateral entorhinal cortex and anterior olfactory nucleus

The LEC is a member of both the olfactory cortex and the hippocampal formation and has strong bidirectional connections between olfactory regions (OB, AON, and PCx), HPC, and parahippocampal areas like the MEC, perirhinal cortex, and postrhinal cortex (Figure 1A) (19, 34, 40). The two primary layer II cell types in LEC are reelin-expressing (RLN+) fan cells in layer IIa and calbindin-expressing (CB+) pyramidal cells in layer IIb (Figure 1B), which together are known to encode odor information (41–43). Most input from the LEC to HPC is via RLN+ fan cells projecting to DG and a subset of CB+ pyramidal cells projecting to CA1, suggesting that the LEC transfers “processed” odor information to HPC (Figures 1B,C) (42). Moreover, CB+ cells project back to OB and PCx (Figure 1B) (41). Due to these strong connections with olfactory areas and HPC, we propose that LEC is an “integrator” of olfactory sensory information and contextual/place information.

Although MEC is more weakly connected to olfactory areas, both MEC and LEC send projections to DG granule cells. This may indicate that HPC can integrate information from both of these areas: spatial information from MEC and contextual information from LEC (44). Thus, it is possible that MEC, despite a lower responsiveness of its cells to odor, is part of the overarching circuitry that positions an animal in their environment and allows them to generate olfactory-spatial memories.

The AON is also proposed to be involved in integrating odor and spatial information due to its direct input from HPC (Figures 1A,C). There is a unique topographic gradient between CA1 neurons in HPC and AON, with the ventral HPC projecting to medial AON, dorsal and intermediate HPC projecting to lateral AON (45). These connections are proposed to play a role in odor contextualization, as hippocampal feedback projections to AON transfer spatiotemporal information, which is then integrated with odor information to form olfactory memory representations (46, 47). Also, inter-hemispheric feedback connections exist from AON to OB (19, 48, 49). These contralateral projections are believed to be relevant for stereosampling, where responses to odors are compared between left and right nostrils to aid in odor localization.

## Neural representations during behavior

Recent experiments have explored how the connectivity detailed above functionally integrates odor and spatial information. Electrophysiological recording and calcium imaging have been the most common methods for observing neural activity during behavior. Some studies employ manipulation of neural activity with techniques including optogenetics and chemogenetics, probing the causal role of specific populations. In parallel, increasingly complex task designs incorporate strategic perturbations or closed loop behavioral interventions.

These studies variously emphasize one or more interrelated questions. How are different odor-spatial relationships represented? How do these representations differ across brain areas? How do these areas communicate with each other? How do representations change across learning? The following section highlights some key recent advances in this research program, especially the use of advanced recording and/or manipulation techniques within cleverly designed behavioral tasks.

### Odors in virtual space

**Figure 2:**
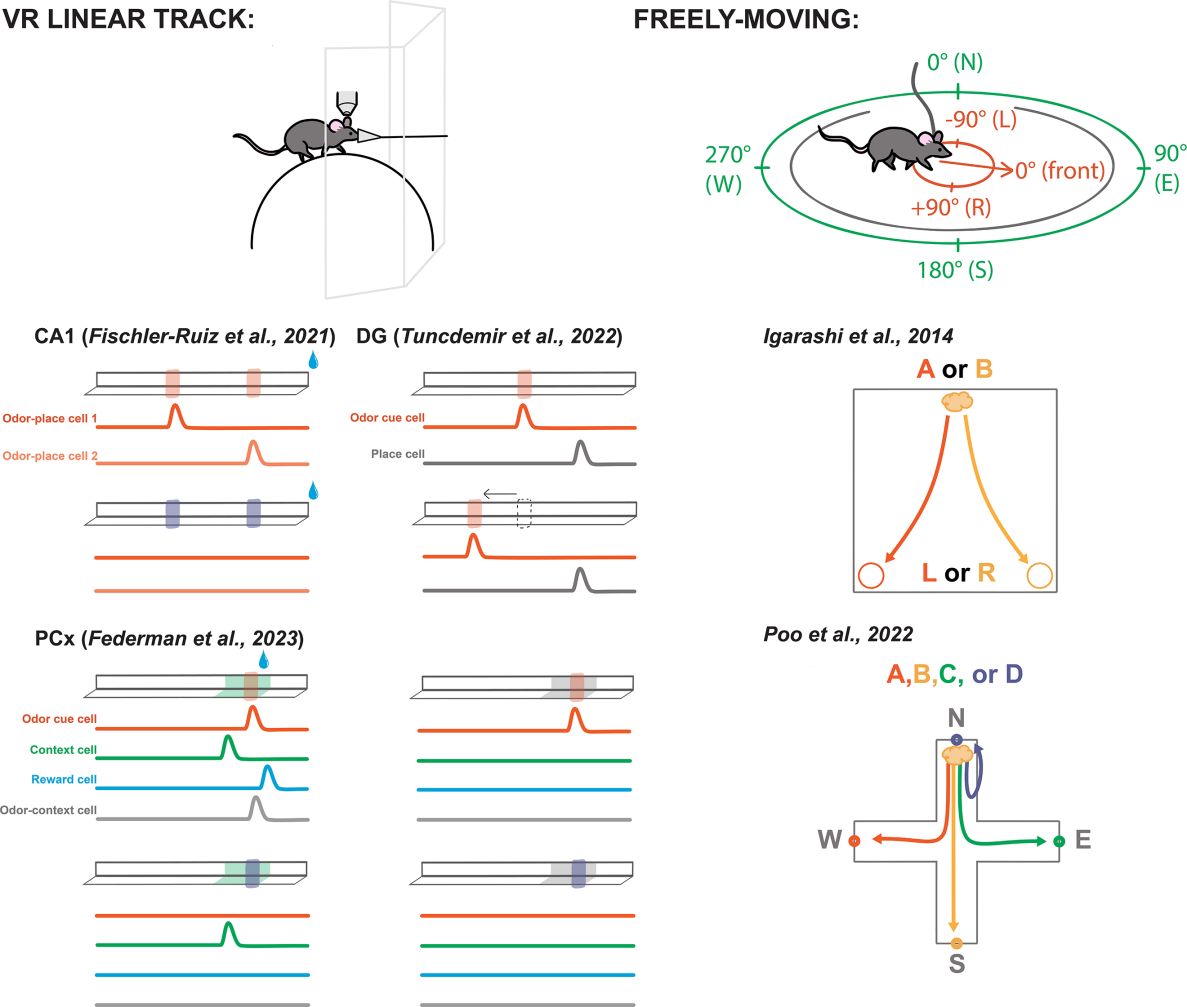
Odor-spatial paradigms for characterizing neural activity across brain areas. Left-top: A typical VR linear track set-up including a rodent head-fixed under a recording device (e.g., two-photon microscope) walking on a sphere or wheel while odor is delivered through a nose cone and visuals are shown on screens. Left-bottom: Simplified depictions of select VR linear tracks featured in this review. Odors are represented by translucent red and purple blocks on the track. Water drops represent reward. Specific visual contexts shown in green and gray. Beneath each track are simplified depictions of featured cell activities from each study. Bump represents an uptick in activity. Cell descriptions are labeled in the first panel for each experiment. Experiments labeled by citation above track depictions. Right-top: Schematic of a freely-moving rodent, allocentric spatial perspective represented in green, egocentric represented in red. Right-bottom: Freely-moving task from Igarashi et al. (50), depicting the correct paths to reward depending on odor presentation, odor A signals a reward at the left, odor B signals reward at the right. Bottom-right: Task from Poo et al. (27) with four odor trials from the same port depicted.

A particularly popular paradigm is the virtual reality (VR) linear track, in which a head-fixed rodent walks on a wheel, sphere, or treadmill, with sensory cues applied to simulate the experience of moving along a straight corridor (Figure 2). Using VR permits presentation of odor and other cues with a level of precision that is difficult or impossible to achieve with freely-moving mice. Additionally, head-fixation enables recording and manipulation techniques that use hardware that is too large for implantation in moving animals. Most of the studies we highlight below use some version of a VR linear track.

### Odor representations in CA1 are driven by spatial information and salience

Radvansky and Dombeck pioneered the use of olfactory stimuli in a VR linear track paradigm (51). Using a custom olfactometer, they precisely controlled odor delivery and presentation of visual cues to mice on a spherical treadmill. They showed that mice anticipated rewards (seen as increased lick rates) at opposite ends of a virtual track even when track position was indicated only by odor gradients. Hippocampal CA1 activity revealed odor-driven place cells in much the same way comparable visual and multisensory tasks show (52, 53).

Similarly, Fischler-Ruiz and colleagues investigated encoding of two localized odor landmarks (Figure 2) (54). They found place cells spanned the track’s length, while noting a heightened density of place cells responsive to the odor landmarks. The vast majority of these place cells were only responsive to one of the two landmarks along the track, meaning that they were not simply responding to the odor cue, but contained an integrated representation of location with the odor. Swapping which odor appeared at the landmarks led place fields to remap, suggesting that the different odor identities were perceived as distinct contexts and that the place coding was intertwined with the odor landmarks.

In a revised paradigm, Radvansky and colleagues trained mice to associate either odor or visual cues with reward (55). They observed substantial recruitment of additional CA1 cells in response to salient cues in both olfactory and visual tasks. In these tasks both olfactory and visual cues were presented, but only one would correspond to reward. Importantly, the cues were not stationary landmarks but moved along the track, eliciting place-like firing leading up to, during, and after the reward relative to the changing location. However, since the odor cue was always spatially tied to reward in the olfactory task, CA1 recruitment could have been due to either odor or reward expectation. Importantly, different populations of cells would fire in relation to the cue-reward depending on the cue modality and task demands. The proportion of visuo-spatial and olfacto-spatial tuned neurons substantially differed between tasks, indicating that the task or reward relevance of sensory input influences hippocampal mapping. Together, this suggests that sensory cues that predict reward overshadow irrelevant cues in CA1 neurons. A further inference is that hippocampal odor-spatial coding is dynamic and adapts to distinct behavioral contexts.

### Dentate gyrus granule cells segregate odor and place coding populations

Other regions of HPC, notably DG, have been implicated in the encoding of odor and spatial information. In this section, we describe activity recorded in DG in response to multiple strategic manipulations to the VR linear track paradigm, summarize functionally identified cell types, and consider connectivity that may be essential to these response profiles.

Tuncdemir and colleagues performed an impressive number of variations on the linear track task, while always delivering reward at random (56). Their experimental setup involved a treadmill equipped with stable textural cues to demarcate the track’s beginning and end. Initially, mice were introduced to an odor cue near the track’s center, consistently positioned relative to the start and end points (Figure 2). Neural activity exhibited place-like tuning along the track’s length, with notable enrichment at the lap cues and the odor landmark, mirroring observations from the previously mentioned experiments in CA1 (54).

Investigating further, they introduced laps where the odor cue was spatially shifted or omitted altogether (Figure 2). This manipulation revealed that the majority of cells initially recruited to the odor landmark would be better classified as cue cells, as their responses shifted or disappeared, respectively. However, some place cells persisted, suggesting that cue manipulation did not induce remapping. With the shifting location of the odor cue, these observations look similar to CA1 neurons described earlier (55), however these odor cues were not tied spatially to the randomly supplied reward.

In a series of additional experiments, the researchers compared coding of cues of different sensory modalities, odor cues in different visual contexts, and cues when presented multiple times across the track (56). They found that cues of all modalities were represented in equivalent ways by mostly separate populations of cue cells, that cue cells remained stable across different contexts, and that cue cells fired reliably to their respective cue whenever presented. In comparison, they found that place cells in the same region were less stable, drifting across days, and remapping in different contexts. Thus, by leveraging simple alterations of the task, the authors were able to differentiate between and characterize two important cell populations in DG: place cells and cue cells. They did note that when an odor cue was consistently presented in the same location, the amplitude of cue cell responses was increased, indicating that there is some level of spatial modulation occurring in DG odor cue cells (56).

Given this interesting dichotomy between cell populations in the DG, one might ask where cue cells and place cells get their information. The authors propose that these separate populations of cue cells and place cells receive projections from LEC and MEC, respectively. LEC is often associated with sensory cues and salience, while MEC is primarily involved in spatial processing (57, 58). Indeed, the robust connection from LEC to DG was already implicated in the transfer of odor information to DG (59, 60). While this particular connection may convey odor identity and salient cues, the LEC has also been shown to output specific salient spatial information (61, 62).

### Encoding of reward locations in LEC and PCx

Recent work delved into the role of the LEC in encoding reward location (61). By introducing uncued reward at a particular location along the track, researchers were able to identify specific pre-reward, reward consumption, and post-reward cells. When the location of this reward changed, these three cell types adjusted to the new location quickly. They also demonstrated that LEC activity was crucial for learning the position of salient cues, as inhibiting the LEC deterred learning of new reward locations.

In a similar reward navigation task, Bowler and colleagues highlighted a dichotomy between LEC and MEC projections to CA1 (62). They found LEC axons encoded both reward and spatial position. When the goal location was changed, the LEC axons appeared to mostly remap. This finding is consistent with CA1 activity described above, where neurons remapped according to moving reward cues (55). In contrast, MEC axons displayed only spatial coding, remapping with context changes, similar to classic place cells described in CA1 (54) and DG (56). Together, these studies illuminate pathways for odor and space integration from the entorhinal cortices to different areas of the HPC, with LEC appearing to exert direct influence over CA1 and remapping in relation to salient cues.

As part of the primary olfactory cortex, PCx is traditionally studied for its role in coding basic odor information such as odor identity and intensity (25, 63–66). Yet robust bidirectional connections with the LEC, AON, and OB suggest some higher associative role for the area (16, 20–22). Thus, Federman and colleagues conducted a comprehensive investigation into the coding capabilities of PCx through a multi-cue multi-context paradigm (67). In a linear track setup using two odor cues and two visually distinct contexts, Federman paired one conjunction of these variables with a reward shortly after the presentation of the odor. Initially, cells responded primarily to the odor cues (Figure 2). However, with continued exposure and learning of the environmental and olfactory associations, more cells responded to each salient feature of the task, including their many combinations. Notably, the authors report both odor specific cells, context specific cells, and conjunctive odor-context cells. This study showed that PCx can represent behaviorally-relevant odor-spatial information (67).

### Freeing behavior: two dimensions and the choice of head-fixation

Thus far, we have summarized studies illustrating diverse coding of odor-spatial information across several key brain areas, with emphasis on the use of a VR linear track. In terms of our hypothetical mouse, we have imagined them foraging for seeds only in a hallway. In addition to this line of VR linear track research, other studies have sought to reap the benefits of head-fixed recording in concert with more complex two-dimensional navigation tasks, for example, employing a spherical ball and a two-dimensional VR world (68) or a floating platform (69). Even in these cases, the use of head-fixation removes important real-world cues. For example, an absence of translational vestibular input and other self-motion cues can produce impairments in two-dimensional place tuning (70, 71). Such limitations motivate parallel studies in more ecologically relevant freely-moving conditions, that we summarize next.

### Spatial coding in different reference frames

Whether in VR or freely moving conditions, including a second dimension opens up consideration of multiple spatial reference frames (Figure 2). For example, we often think of space as a fixed map of locations depicted in relation to each other. This perspective is called “allocentric,” for example with directions referred to as north, south, east, and west. Simultaneously, we can experience the world in an “egocentric” perspective, using directions to locations such as front, back, left, and right that depend on our place in the world. While peripheral sensory input is inherently egocentric, both perspectives are used for encoding information in the brain, and it is thought that the allocentric perspective is dominant by the time sensory information gets to HPC (72).

Early research into spatial coding was approached primarily from an allocentric perspective, including discoveries of place, grid, and head-direction cells (73–77). Recently, however, studies have identified more egocentric coding throughout the brain. Wang and colleagues characterized spatial coding in the LEC, finding egocentric coding of several aspects of the environment that would be difficult to recognize in a linear track task (78). By leveraging natural exploratory behavior in mice, they discovered cells tuned to boundaries, items, and goal locations. Together, these findings suggest that the peri-reward cells in LEC discussed earlier could be encoding egocentric coordinates of the reward location (61).

Part of the difficulty in identifying egocentric coding is that cells could be tuned to any of a variety of locations or items in an environment, and tasks must be designed specifically to dissociate different possible coordinate-systems. For example, Igarashi and colleagues trained rats to discriminate two different odors associated with corresponding reward locations (Figure 2) (50). They used egocentric terms “left” and “right” to describe reward locations, but the task design could equivalently be described in allocentric terms such as “East” and “West.” The goal of their study was not to explore different spatial frameworks, so they did not include additional spatial complexity, such as rotating or flipping the environment between trials or sessions. However, this study revealed synchrony between LEC and CA1 during odor-place association, which we will discuss further below.

In order to differentiate between allocentric and egocentric coding, it is necessary to examine neural activity during more spatially complex behaviors. Poo and colleagues trained rats to associate four different odors with reward at each of four different ports in a plus shaped arena (Figure 2) (27). Notably, the trial initiation port also changed locations between each trial, requiring the association of each odor to an allocentric place (e.g., North reward port) rather than to an egocentric action (e.g., Left turn to reward port). Interestingly, they found place-like neural tuning not only in CA1. Owing to the clever task design, they further were able to dissociate odor identity from odor delivery location and found both odor selective cells and place-like cells in posterior PCx. They were also able to determine that pPCx spatial coding was allocentric, something that had not yet been described. These spatial PCx cells were concentrated at odor/reward delivery ports indicating that the posterior PCx may represent locations relevant to olfactory task demands.

## Learning integrated odor-space representations

An animal is not born with a cognitive map of the world. Representations must be learned through experience and updated as the world changes. The olfactory-hippocampal circuit is especially equipped to facilitate learning, but there are still many unknowns in how representations change across these brain areas to encode new information. So far, we have touched only briefly on studies that investigate changes in neuronal activity during learning. Here we elaborate these findings and propose connections between them.

### Inter-areal synchronization during associative learning

Neural activity synchronization has long been associated with learning and memory and navigation (79, 80). Studies have found synchronization between key areas during olfactory-spatial task performance (27, 50). Igarashi and colleagues demonstrated synchronization of the CA1 and LEC during the presentation of odor cues in their left–right odor association task (50). Poo and colleagues found PCx spatial neurons were synchronized with CA1 firing (27). Taken together, these studies suggest that all three areas are synchronized during odor-place association, and further that top-down connections to PCx may be responsible for its spatial coding. Meanwhile, odor tuned cells in PCx tended to be synchronized to the sniff cycle, suggesting that these cells were more directly influenced by OB (27).

Igarashi and colleagues also observed synchrony evolve over multiple days as they added new odors, forcing rats to learn new odor-spatial associations; as rats learned to perform with higher accuracy, the synchronization of the LEC and CA1 also increased (50). This phenomenon may reflect Hebbian strengthening of synapses (81, 82). Indeed, the authors found that the selectivity of odor representations in LEC and CA1 was highly correlated with this inter-areal coupling, and suggested that this coupling may be responsible for the formation of associative representations in both areas.

### Intrinsic and learned representations

Complementary studies distinguish representations that seem to be intrinsic to a given brain area from new representations recruited through learning. For example, while place cells in CA1 appear almost instantly upon introduction to a new environment (83) [with some nuances to consider (77)], the appearance of odor landmark tuning was highly correlated with behavioral indications that animals learned the significance of these environmental cues (54). Similarly, odor was strongly represented in CA1 when it predicted reward, but not when another cue was more important to the task (55). Together, these findings support that CA1 forms representations of salient odors in space through associative learning. Anterior PCx seems to mirror this process, with initial coding only of odor, followed by recruitment of more contextual, spatial, and conjunctive odor-context cells with learning (67). Fully trained rats show both spatial and odor representations in posterior PCx. It is unclear whether posterior PCx automatically represents spatial information in a new environment, but the concentration of spatial cells around behaviorally relevant locations suggests that they are recruited through learning (27). Given synchrony between CA1 and PCx and the diverse representations that form in both areas over the course of learning, it appears that these areas may be sharing information to encode salient odor-spatial associations.

### A role for LEC in mediating odor-place integration across areas

How does the PCx-HPC circuit know what information to integrate? The LEC is particularly well poised to manage this process. With bidirectional connections to both CA1 and PCx, the LEC stands in position to gate information between them.

Indeed, the LEC has been shown to be important in both spatial and olfactory learning. For example, one study demonstrated that the LEC is required for learning new reward locations (61), while others show that inhibiting LEC pathways to either CA1 or DG impairs new olfactory association learning (42, 60). Interestingly, these studies noted that learning was impaired, while pre-learned associations were not affected. This suggests that LEC plays a specific role in encoding new information, especially odor and spatial associations, thus we hypothesize that LEC acts as a key gateway for pairing these salient associations by inducing multi-areal oscillations and forming complementary representations in HPC and PCx.

Lee and colleagues go farther, identifying dopaminergic inputs to the LEC as key to learning new associations in an odor discrimination task (60). Dopamine is one possible trigger to identify novel salient information. The literature on dopamine is vast, and the idea that it may signal novel information underlying learning is key (84–86). That said, the LEC is a highly connected area, and yet unidentified connections may also be important for triggering the process of forming new odor-spatial associations (87).

Interestingly, the LEC sends the bulk of its output projections to DG. Both in LEC fan cells and the DG cells to which they project, odor exposure elicits activity without prior learning (59, 88). As explained earlier, DG maintains separate populations of place cells and cue cells throughout several manipulations, with little evidence of integration (56). This sparsity may be important to differentiate between contexts and distinct episodes (44). During an odorant discrimination task, representations in DG get more sparse and more specific to the odor identities; further, the connection between LEC and DG is important in learning these representations (59). Thus, DG may be responsible for cueing specific contexts, and signaling CA1 to represent salient context-dependent information.

Taken together, these observations may suggest that learning of odor-place associations cause CA1 and PCx to become more tuned to both odor and spatial information, perhaps owing to correlated firing and strengthened connections with each other, mediated by LEC. Simultaneously, DG forms a sparse representation of contextual information that corresponds to learned associations in CA1, for example making it possible for odors to hold different spatial associations in different contexts. We propose that associations between CA1 and PCx are gated by the LEC, which then entrains the CA1-LEC-PCx network in order to tie PCx and CA1 representations together.

**Figure 3:**
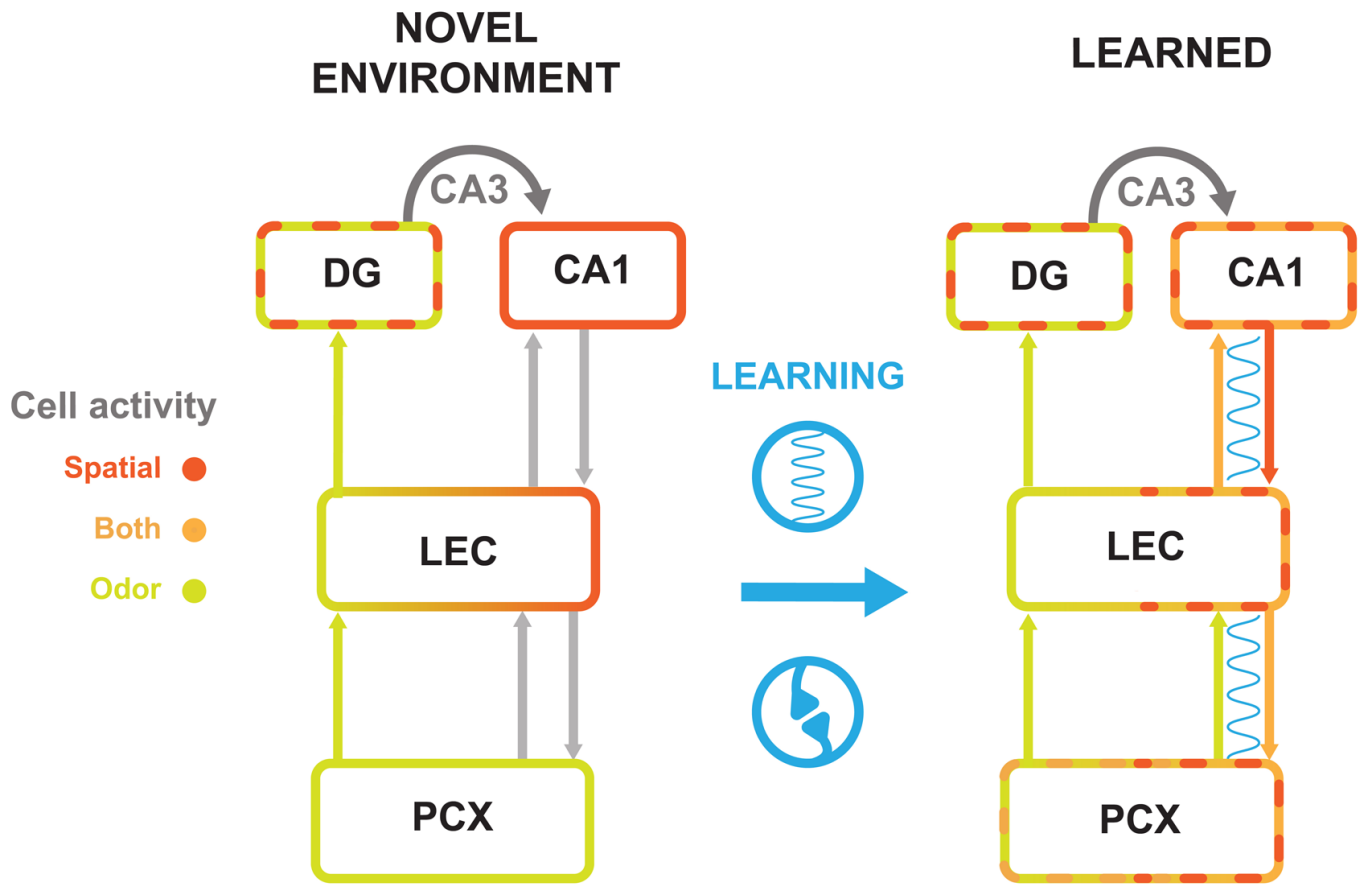
Cell activity changes with olfactory-spatial learning across the PCx-HPC circuit. Summary model of the flow of information along the HPC-LEC-PCx pathway. Schematics represent circuitry in a novel environment (left) and after an association is learned (right). Synchrony between areas is represented by a blue wavy line. Arrows between areas represent projections from cell populations with indicated tuning. Spatial (scarlet), odor (chartreuse), and conjunctive cell (orange) activity are represented by colored outlines. Learning is represented in blue between circuitry models, with synchrony and synaptic plasticity icons to symbolize the changes made between the two timepoints. Gray arrows to and from LEC represent connections that exist but are not thought to be synchronized before learning. Dashed boundaries represent cell populations that contain more than one firing type; for example, the DG exhibits odor cue cells and place cells even in novel environments, while the CA1 in the learned condition contains place cells and odor-place cells.

Under this model (Figure 3), after introduction to a novel environment, PCx would primarily represent odor and CA1 space. As the animal recognized that an odor cue always preceded a reward location, synchronization of PCx, LEC, and CA1 would increase. This synchronization would signal inter-areal communication that strengthened their connections. These connections would then lead to the emergence of odor-evoked ensembles in CA1 and spatially tuned ensembles in PCx. Given that both areas would then contain the integrated odor-spatial associations, this would also account for the observation that LEC is needed for learning new associations, but not recalling ones previously formed (42, 60, 61).

## Future perspective

Acquiring data throughout the learning process is challenging. However, emerging tools are making it possible to acquire, store, analyze, and share the vast and multifaceted data produced by olfactory-spatial learning experiments. New indicators and better imaging technologies are rapidly being developed (89–92), while the channel count of electrophysiological recording has dramatically increased (93, 94). Larger datasets impose additional data management requirements, relying on improvements in storage size and decreasing costs (95, 96), and data standards such as NeuroData Without Borders (97). Videographic analysis of behavior has been tremendously advanced by automated tools such as DeepLabCut (98) and MoSeq (99), and an array of post-processing analysis pipelines, e.g., VAME (100), B-Soid (101), DeepPoseKit (102), or Keypoint-MoSeq (103).

Together, these technologies will support novel and advanced investigation of how sensory experience in a dynamic environment shapes the synaptic, cellular, and circuit mechanisms enabling behaviors relying on the integration of odor and space.

## Author contributions

OM: Conceptualization, Visualization, Writing – original draft, Writing – review & editing. NK: Conceptualization, Visualization, Writing – original draft, Writing – review & editing. JR: Conceptualization, Writing – original draft, Writing – review & editing. AF: Conceptualization, Writing – original draft, Writing – review & editing.
